# The use of phosphotriesterase in the synthesis of enantiomerically pure ProTide prodrugs

**DOI:** 10.1016/j.cbi.2025.111597

**Published:** 2025-06-06

**Authors:** Andrew N. Bigley, Frank M. Raushel

**Affiliations:** a Department of Chemistry and Physics, Southwestern Oklahoma State University, Weatherford, OK, 73096, USA; b Department of Chemistry, Texas A&M University, College Station, TX, 77843, USA

**Keywords:** ProTide, Phosphotriesterase, Prodrugs, Chemoenzymatic synthesis, Antiviral

## Abstract

Outbreaks of viral diseases, such as COVID-19, and chronic viral diseases, such as HIV and hepatitis, have highlighted the need to develop antiviral medications. ProTide nucleotide analogs such as Remdesivir and Sofosbuvir have become an important class of antivirals. The ProTides are phosphonamidate prodrugs, which contain an alanine ester and a phenyl group esterified to a chiral phosphorus of a nucleotide analog. The resulting triester effectively masks the charge on the phosphate moiety to facilitate entry into the cell and are much more effective than the corresponding nucleoside analogs. Once in the cell, the ProTides require activation by cellular enzymes to remove the masking groups on the phosphorus. The activation in the cell is dependent on the stereochemistry of the phosphorus center with the effectiveness of a given isomer differing between tissue types. The ProTides are produced as single isomers at the phosphorus center by chiral chromatography or selective crystallization, but in many cases only a single isomer can be produced, potentially limiting the effectiveness of the ProTides. The phosphotriesterase (PTE) from *Brevundimonas diminuta* is well known for its ability to selectively hydrolyze chiral phosphotriesters. The extensive directed evolution of PTE has led to the identification of variants that can selectively hydrolyze the phosphonamidate precursor of the ProTides, allowing the preparation of optically pure ProTides. Importantly, the variant In1W-PTE allows the isolation of the pure *R*_P_-isomer while G60A-PTE and W131M-PTE allow the isolation of the pure *S*_P_-isomer, thereby facilitating the efficient preparation of either isomer of the final ProTide.

## 1. Background

**Fig. 1.**
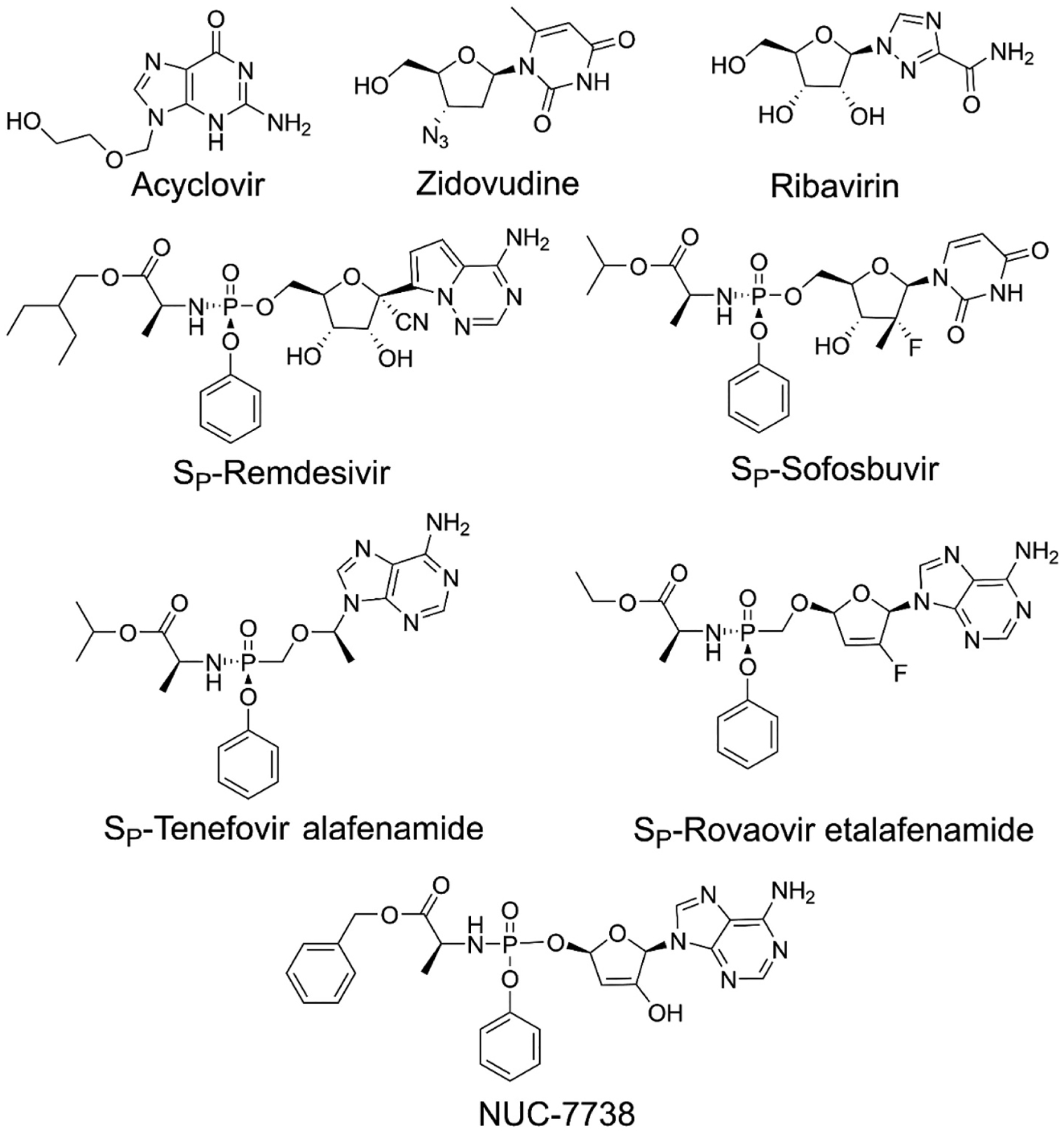
Structures of nucleoside analog prodrugs and nucleotide phosphonamidate prodrugs (ProTides).

Epidemics of viral diseases have become defining events in the modern world. Among the events that have had profound effects on modern life are the HIV pandemic of the 1980s and 1990s, the Ebola outbreak in Africa in 2014, the SARS and MERS outbreaks in China and the Middle East in 2003 and 2012, respectively, and the COVID-19 pandemic of 2019–2023 [1–4]. These major world events are in addition to the tens of millions of cases of hepatitis and hundreds of millions of cases of herpes that are chronic burdens on the human population [5–7]. Fortunately there has been much work to develop antiviral medications with more than ninety FDA approved treatments having been brought to market over the last 60 years. (Reviewed in ref 8) A significant number of these compounds are nucleoside analogs that target viral DNA polymerases, reverse transcriptase, and RNA polymerases to disrupt viral genome replication [8]. The initial nucleoside analogs were nonspecific molecules that inhibited both viral and host enzymes [8,9]. All nucleoside analogs are prodrugs that require conversion to the corresponding triphosphates to be active. The discovery of compounds such as Acyclovir, which is specifically activated by a viral kinase, allowed for the direct targeting of infected cells with minimal effect on healthy cells leading it to become the “gold standard” for herpes simplex virus infection (Fig. 1) [9]. Other compounds such as Zidovudine (AZT), used to treat HIV, and Ribavirin, used to treat hepatitis C and respiratory syncytial virus (RSV), specifically target the viral polymerase over the host polymerases allowing direct activity against virus replication and gene experession [8,10,11].

### 1.1. Limitations of nucleoside and nucleotide prodrugs

The nucleoside prodrugs have certainly seen clinical success, but they have intrinsic limitations due to low bioavailability, poor cellular permeability, development of resistance, and toxicity. Nucleoside prodrugs must be phosphorylated by cellular or viral kinases to the triphosphate form to have the requisite activity. This phosphorylation can be inefficient and known resistance mechanisms involve down regulation of kinase expression [12–14]. The nucleoside prodrugs are polar compounds that generally enter the cell via active transport limiting the accumulation in cell [14]. These limitations can be overcome in part by the use of nucleotide monophosphate analogs rather than nucleosides, but the poor stability of the phosphorus bonds in vivo and poor cellular penetration were found to be problematic [15]. The first attempt to address these issues was the development of phosphonate prodrugs such as Tenofovir, which is FDA approved for hepatitis B [16]. The introduction of the P–CH_2_ bond doubled the circulation half-life of the compound, but the problems of bioavailability and cell penetration remained.

### 1.2. Development of phosphonamidate nucleoside prodrugs

The novel approach pioneered by Dr. Chris McGuigan of Cardiff University was to convert the phosphate group to a nitrogen containing triester to mask the charge [17]. The latest versions of these compounds as exemplified by Remdesivir and Sofosbuvir, generally contain a nitrogen-phosphorus bond linking the phosphate to an l-alanine that has the acid group esterified. The remaining phosphorus oxygens are esterified to the nucleoside and to a phenol group to completely mask the charge on the phosphate (Fig. 1). This masking scheme, commonly referred to as ProTides, is highly effective at delivering active nucleotides to the cell. The ProTide version of Tenofovir is 1000-fold more effective than the parent nucleoside at inhibiting HIV reverse transcriptase [18]. Sofosbuvir is now the recommended first line treatment for hepatitis C infection. The parent nucleoside of Sofosbuvir does not display any antiviral activity due to the inability to be phosphorylated in the cell [19]. The ProTide Remdesivir, which is effective against Ebola, RSV, hepatitis C, SARS, MERS and approved for COVID-19, is at least 100-fold more active than its parent nucleoside [20]. Additional ProTides include Rovafovir etalafenamide, which is a phosphonate under development as an HIV inhibitor and NUC-7738, which is being tested as an antitumor agent to overcome resistances due to adenosine deaminase and adenosine kinase deficiency [21,22].

## 2. Intracellular activation of ProTides

**Fig. 2.**
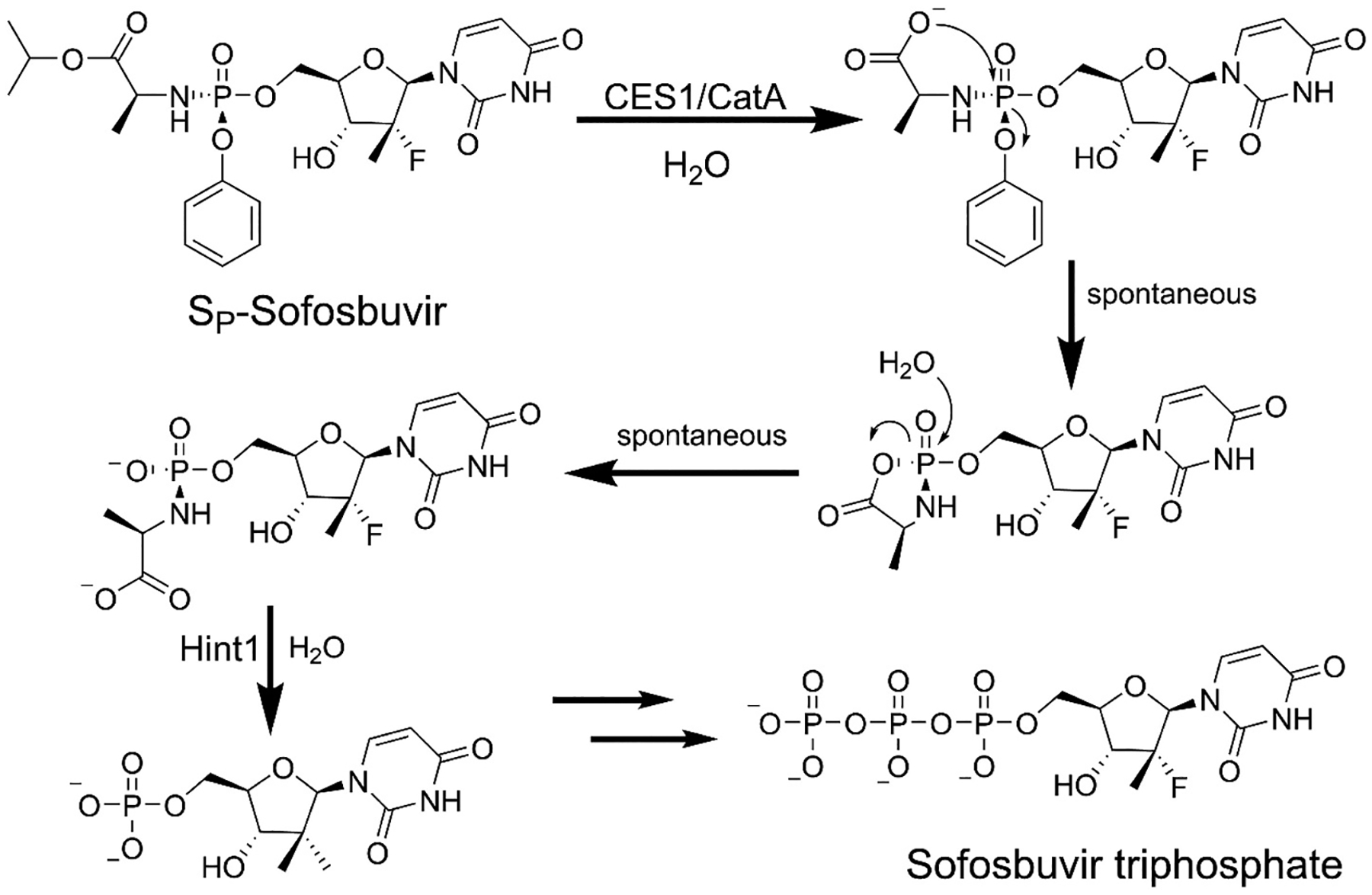
Activation of S_P_-Sofosbuvir to Sofosbuvir triphosphate in the cell.

ProTides require activation intracellularly by sequential cleavage of the amino acid ester bond and phosphoester bond followed by phosphorylation to the nucleoside triphosphate (Fig. 2). The first bond to be cleaved is the amino acid ester by either carboxylate esterase 1 (CES1) or Cathepsin A (CatA) [19,23–27]. The liberated carboxylase group then makes a nucleophilic attack on the phosphorus center liberating the phenol group and forming the cyclic phosphonamidate intermediate [28]. The cyclic intermediate is then hydrolyzed to yield the alanine ester. It was initially unclear if the spontaneous attack on the cyclic phosphonamidate came at the carboxyl group or at the phosphorus center, but it has now been demonstrated that the attack comes exclusively at the phosphorus center [29].

### 2.1. Stereochemical dependence of ProTide activation

As has been seen with prodrugs such as chlorambucil, the stereochemistry of chiral centers in prodrugs can significantly impact the biological activity of the compound even in cases where the stereocenter is lost in the active compound [30]. The ProTides contain stereocenters at both the α-carbon of the amino acid and at the phosphorus. The amino acid is cleaved in the activation, and neither the cleaved nucleotide prodrug nor the active nucleoside triphosphate for the ProTides contain a chiral phosphorus [16]. However, the stereochemistry of both the amino acid and the phosphorus significantly impact the efficacy of the prodrug [18,25,27]. The alanine typically used in the ProTides is the l-isomer. Studies with Sofosbuvir showed incorporation of d-alanine resulted in more than 100-fold reduction in effect against hepatitis C [31]. Similarly the incorporation of d-alanine in Remdesivir resulted in approximately 10-fold less activity against Ebola [20]. While the effect of the stereochemistry of the α-carbon is fairly straightforward with the strong preference for the L-conformation, the effect of the stereochemistry at the phosphorus center is more complex. The S_P_-isomer of Sofosbuvir is 18-fold more effective against hepatitis C [31]. The S_P_-isomer of Tenofovir alafenamide is about 12-fold more effective against HIV than the R_P_-isomer [18]. However, MK-3682, which was developed against hepatitis C showed approximately 20-fold faster activation than the S_P_-isomer [11]. This extensive study with ProTides showed that there can be little difference between the R_P_- and S_P_-isomers of a given ProTide [11]. In the case of Remdesivir both the R_P_- and S_P_-isomers were found to be active antivirals with the efficacy depending on the tissue being targeted [20].

### 2.2. Tissue targeting of ProTides

One of consequences of the stereochemical dependence of the ProTide activation is that the ProTides accumulate in tissues that are best able to activate the compounds. Once the ester bond is cleaved the cyclization and loss of the phenol group is spontaneous trapping the ProTide in the cell. This has allowed for the ProTides to be targeted to specific tissues. Both Tenofovir alafenamide and Sofosbuvir are known to accumulate in hepatic tissues [11,32]. Tenofovir alafenamide is also known to accumulate in lymphatic tissues and peripheral blood mononuclear cells, which is of importance to the application as an anti-HIV medication [18]. Further studies have shown that this ability to accumulate in specific tissues is due to the tissue specific expression of the enzymes that catalyze the first step in activation in particular CES1 and CatA [24,27,33]. It is well known that both CES1 and CatA are stereospecific with opposite preferences. [26] CatA has a fairly wide tissue distribution and a strong preference to the S_P_-isomers of the ProTides, however, the expression levels of CatA are known to vary widely between individuals [34]. CES1 has a preference for the R_P_-isomers of the ProTides and a more limited tissue distribution, but CES1 is the major esterase in some tissues [26,34].

## 3. Improvements in the synthesis of ProTides

**Fig. 3.**
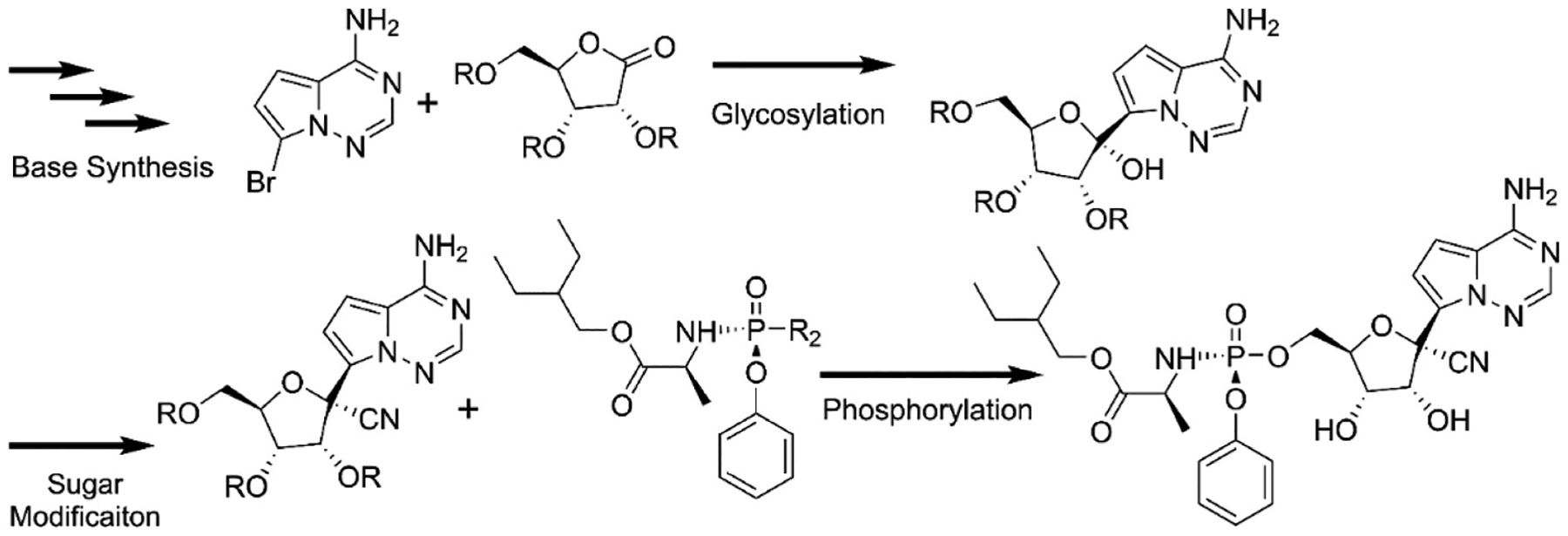
General scheme of the synthesis for the ProTide Remdesivir. The substituent R is generally a benzyl protecting group. R_2_ is Cl, *p*-nitrophenol, or pentafluorophenol.

**Fig. 4.**
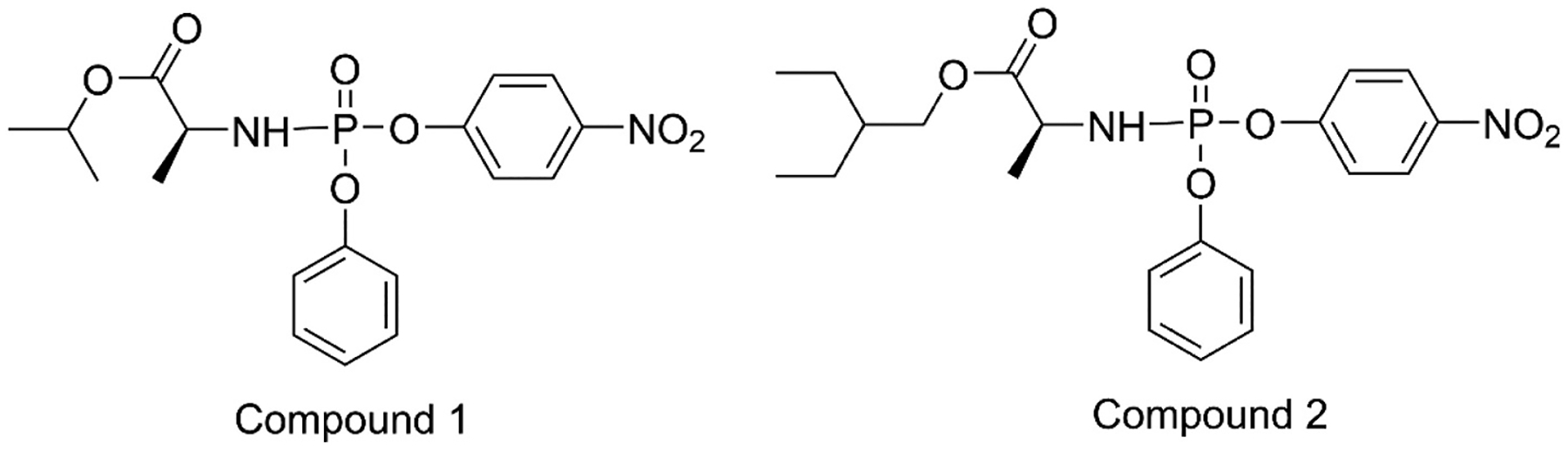
*p*-Nitrophenyl phosphonamidate precursors to Sofosbuvir (**1**) and Remdesivir (**2**).

One of the consequences of the 2020 COVID-19 pandemic was the growing awareness of the difficulties in dramatically increasing production pharmaceuticals including the ProTide prodrugs like Remdesivir [35]. The strategy of ProTide synthesis follows the general route of nucleobase construction followed by glycosylation where the base is attached to the ribose ring. That is followed by modification of the ribose and then finally attachment of the preformed phosphoester containing the phosphate esterified to the amino acid ester and the phenol group (Fig. 3). The exact chemical pathway will by necessity vary from one prodrug to another. Remdesivir, which contains an unnatural base requires the synthesis of the base prior to glycosylation [35,36]. Other prodrugs such as Sofosbuvir or Rovafovir etalafenamide do not have modified bases and synthesis can generally start with the natural nucleobase or nucleoside and in some cases the modification of the sugar is done prior to addition of the base [31,37,38]. Significant work has been done on optimizing the three fragments of the ProTides as well as the reactions to couple them together [35–43]. In particular, work has been done to optimize the synthesis of the nucleobase of Remdesivir for industrial scales [35,36]. Additional work has been done to optimize the glycosylation step for Remdesivir and Adafosbuvir, as well as the synthesis and modification of the glycosyl groups of Remdesivir, Rovafovir and Sofosbuvir [37–39,42,44]. The optimization of the phosphorylation has proven more challenging. The phosphonamidate ester group is synthesized prior to the attachment to the nucleoside. The original protocols started with phosphorus trichloride and coupled the amino acid ester and phenyl group resulting in the phosphorochloridate precursor as a racemic mixture at the phosphorus center. Coupling to the nucleoside consequently resulted in a mixture of the diastereomers at the phosphorus center. Generally, the development of ProTides requires testing of the efficacy of the individual isomers, which must be isolated either by chiral chromatography or selective crystallization [20,31]. These methods are both time consuming and generally inefficient. Further efforts to improve the phosphorylation to achieve single isomers at the phosphorus center involved conversion of the phosphorochloridate precursor to a phosphotriester with either p-nitrophenyl or pentafluorophenol as the third ester group (Fig. 4) [45,46]. An individual isomer of the precursor can then be isolated, generally by selective crystallography, prior to coupling to the nucleoside to yield a single isomer of the ProTide. The coupling to the nucleoside is accomplished by a displacement reaction in the presence of a base where the p-nitrophenyl or pentafluorophenyl group serves as a labile leaving group [41,45,46]. The selective formation of the single isomer is further enhanced by the inclusion of a Lewis acid such as t-buMgCl or Me_2_AlCl. Unfortunately, these methods still typically require 2 or more equivalents of the phosphonamidate precursor to obtain high yields of the final product and result in undesired side products. In many cases only one of the two isomers can be made in significant quantities. As discussed above, the active ProTides such as Sofosbuvir, Adafosbuvir and Remdesivir are known to be tissue specific due to the enzymes required for in in vivo activation. The inability to produce significant quantities of both isomers potentially limits the broader application of these antivirals [20,23,26,27,34].

## 4. The use of phosphotriesterase in the production of ProTides

There has been significant interest in the use of biocatalysts in the production of industrial and pharmaceutical chemicals [47]. This interest also extends to the development of biosynthetic routes of antiviral compounds as well [48]. This has included the use of phosphorylase enzymes in the glycosylation step of Ribavirin and other nucleoside mimics [49]. Enzymes are a particularly good option when specific stereochemistry is required in the formation of pharmaceuticals. Enzymes have been successfully utilized to achieve the correct stereochemistry in the glycosyl portions of both Tenofovir and Sofosbuvir [50,51]. The enzyme phosphotriesterase (PTE) from Brevundimonas diminuta (formerly Pseudomonas diminuta) has been successfully utilized to yield the pure *R*_P_-isomer of Remdesivir.

### 4.1. Phosphotriesterase

**Fig. 5.**
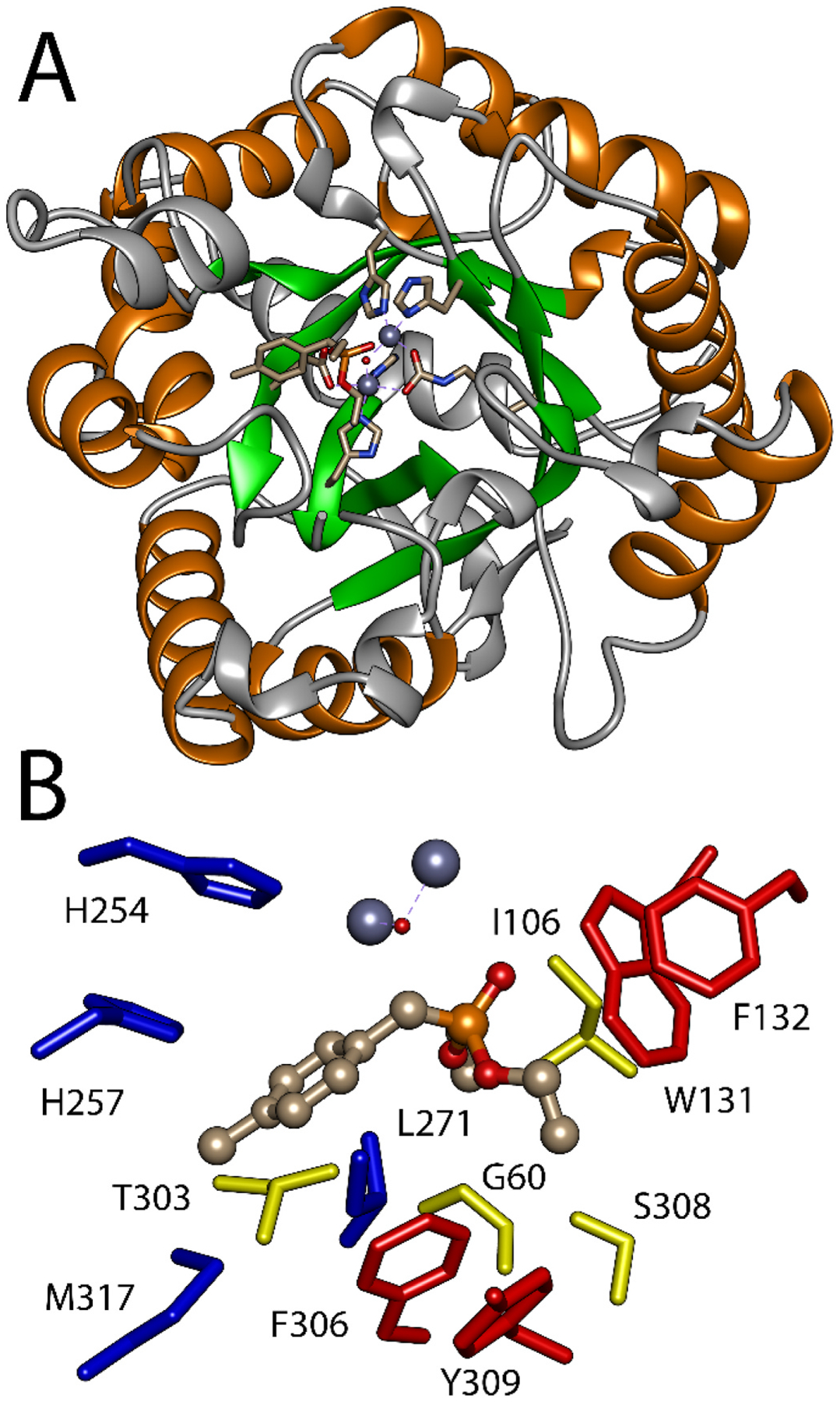
Crystallographic structure of PTE enzyme with the substrate analog diethyl phenylphosphonate bound. A) (αβ)8 structure of PTE barrel with binuclear metal center embedded at C-terminal end of central β-strands. B) Substrate binding pockets of PTE. Leaving group pocket residues W131, F132, F306, and Y309 are shown in red. Large group pocket residues H254, H257, L271, and M317 are shown in blue. Small group pocket residues G60, I106, T303, and S308 are shown in yellow. Structure is from PDB:1DPM. Figure was prepared with USCF Chimera.

The phosphonamidate precursor of the ProTides is effectively a phosphotriester. PTE is well known for its ability to stereo-selectively hydrolyze phosphotriesters [52,53]. PTE was originally identified for its ability to hydrolyze organophosphate insecticides and is one of the few enzymes effective with both phosphotriesters and thiophosphotriesters [54]. PTE was later found to demonstrate strong stereoselectivity with chiral phosphotriesters as well as phosphonodiesters and phosphinate esters [55,56]. The structure of PTE is a distorted (α/β)_8_ TIM barrel (Fig. 5A) [57]. The active site of PTE contains a binuclear metal center ligated to the C-terminal ends of the central β-sheets. The substrate binding site is above the metal center. Substrates bind with ligation of the phosphoryl oxygen to the metal center and the three ester groups placed into three distinct binding pockets made up of the loops that connect β-sheets to the following α-helix in the core structure. The residues that contribute to the specificity for the leaving group (leaving group pocket), the small ester group (small group pocket), and large ester group (large group pocket) have been experimentally identified [58]. The leaving group pocket is lined by residues W131, F132, F306 and Y309 (Fig. 5B). The large group pocket is lined by residues H254, H275, L271, and M317. The small group pocket is a cavity in the enzyme lined by residues G60, I106, L303, and S308.

### 4.2. Directed evolution of PTE

The ability of PTE to hydrolyze organophosphate nerve agents and insecticides led to intensive efforts to further evolve the enzyme [59]. Against its best substrates the reaction by PTE is diffusion limited (k_cat_/K_m_ ~ 10^8^ M^−1^s^−1^) [60]. The wild-type enzyme prefers the S_P_-isomers of chiral phosphotriesters with the activity against the R_P_-isomers being significantly reduced for many compounds (k_cat_/K_m_ ~10^6^ M^−1^s^−1^) [53]. Evolving PTE for the degradation of the organophosphate nerve agents required enhancement of the hydrolysis of the R_P_-isomers. This work led to a large number of PTE variants with varying substrate specificity as well as enhanced, relaxed, and reversed stereospecificity [55,61,62]. Variants of PTE that have been of particular interest included the small group pocket variant G60A which results in dramatic enhancement of the wild-type preference for the S_P_-isomer. Wild-type PTE demonstrates about a 20-fold selectivity for the S_P_-isomer when the large and small groups are phenyl and ethyl respectively. G60A demonstrates 11,000-fold selectivity for the S_P_-isomer of this compound [55]. Other variants like I106G and F257Y relax the stereoselectivity of PTE. Variants that combine mutations in both the large and small group pockets like H257Y/L303T and I106G/F132G/H257Y can reverse the stereoselectivity of the wild-type enzyme. Work to evolve PTE for organophosphate nerve agent decontamination has led to multiple variants that can effectively be used to selectively hydrolyze single isomers of nerve agents and their analogs [63,64]. These variants have also been effectively utilized to synthesize chiral organophosphate compounds from prochiral precursors [65,66].

### 4.3. The use of PTE in the synthesis of ProTides

**Fig. 6.**
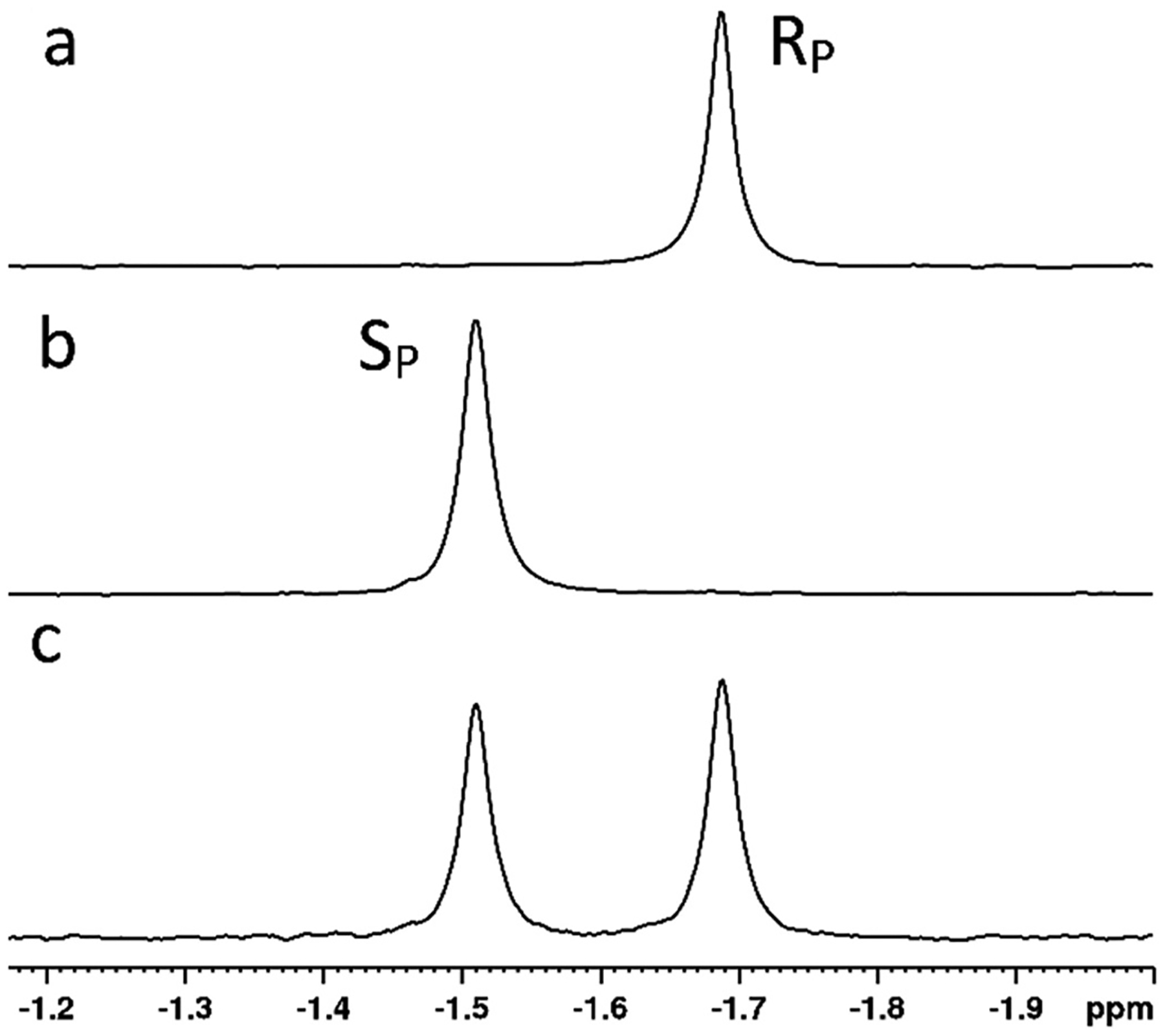
^31^P NMR spectra of compound **1**. The *S*_P_-isomer resonates at −1.52 ppm and the *R*_P_-isomer resonates at −1.69 ppm. a) racemic mixture of isomers. b) Isolated R_P_-isomer after selective hydrolysis by In1W-PTE. c) Isolated S_P_-isomer after selective hydrolysis by G60A-PTE. Data reproduced from reference 67 with permission.

The insecticide substrates of PTE generally contain a dimethyl or diethyl phosphorus center, while the nerve agents contain a methyl phosphonate. By contrast the phosphonamidate precursors to the ProTides are quite large with the phenyl and amino acid ester as the side groups. There is also considerable difference in the amino acid esters utilized in the different ProTides. While they all use l-alanine as the amino acid, Rovafovir etalafenamide uses an ethyl ester, Sofosbuvir uses an isopropyl ester, Remdesivir uses 2-ethyl butyl ester, and NUC-7738, which was developed as an anticancer compound uses a benzyl ester [19–22]. When tested with the p-nitrophenyl precursor of Sofosbuvir (compound **1**, Fig. 4), wild-type PTE showed good activity (k_cat_/K_m_ = 4.7 × 10^4^ M^−1^s^−1^) but very little stereoselectivity [67]. Subsequently compound **1** was tested against a library of PTE variants of differing substrate preferences and stereoselectivity that had been developed for hydrolysis of G-type and V-type nerve agents [64,67–69]. T he enhanced stereoselective variant G60A was found have significantly reduced activity against compound **1** (k_cat_/K_m_ = 2.8 × 10^3^ M^−1^s^−1^) but showed approximately 100-fold preference for the hydrolysis of the R_P_-isomer. It should be noted that the R and S designations of stereochemistry of the phosphonamidate ProTides as well as their precursors is opposite to that of the phosphate triesters due to the lower priority of the nitrogen bond, so the preference of the G60A variant was as expected. The strong stereoselectivity of G60A towards the R_P_-isomer allowed the effective isolation of the S_P_-isomer with the *R*_P_-isomer reduced to levels not detectable by ^31^P NMR (Fig. 6).

**Fig. 7.**
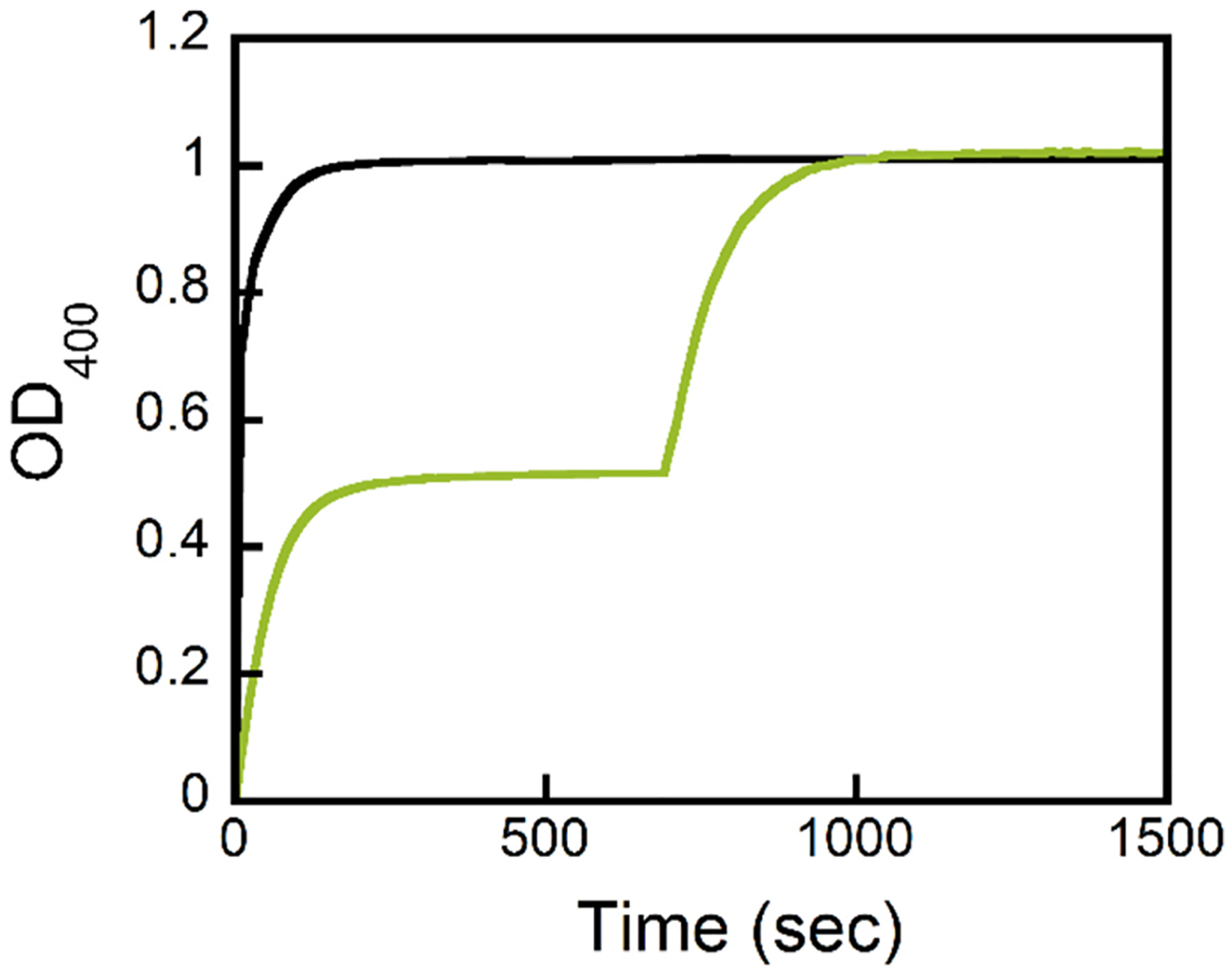
Complementation assay showing G60A-PTE and In1W-PTE hydrolyze opposite isomers of compound **1**. Chemical hydrolysis of 60 μM compound **1** by 1 M KOH (Black). Enzymatic hydrolysis of 60 μM compound 1 (Green) initiated by the addition of 7.2 nM In1W-PTE and then 1.0 μM G60A PTE was added at 700 s. Data reproduced with permission from reference 67.

The best variant against the *S*_P_-isomer of compound **1** was a previously uncharacterized variant (In1W) with a 10 amino acid insertion in the active site loop which connects β-strand 7 to α-helix 7. In1W-PTE was found to have strong activity (*k*_cat_/K_m_ = 2.3 × 10^5^ M^−1^s^−1^) against only a single isomer of compound **1**. Complementation assays with G60A-PTE showed that In1W-PTE prefers the *S*_P_-isomer of compound **1** (Fig. 7). The variant In1W-PTE was effectively found to only hydrolyze the S_P_-isomer of compound **1** with more than a 1000-fold selectivity. Using selective hydrolysis allowed the isolation of ***R***_**P**_**-1** with levels of the S_P_-isomer undetectable by ^31^P NMR (Fig. 6b). The kinetic constants are summarized in Table 1.

#### 4.3.1. Use of PTE in the synthesis of Remdesivir

**Fig. 8.**
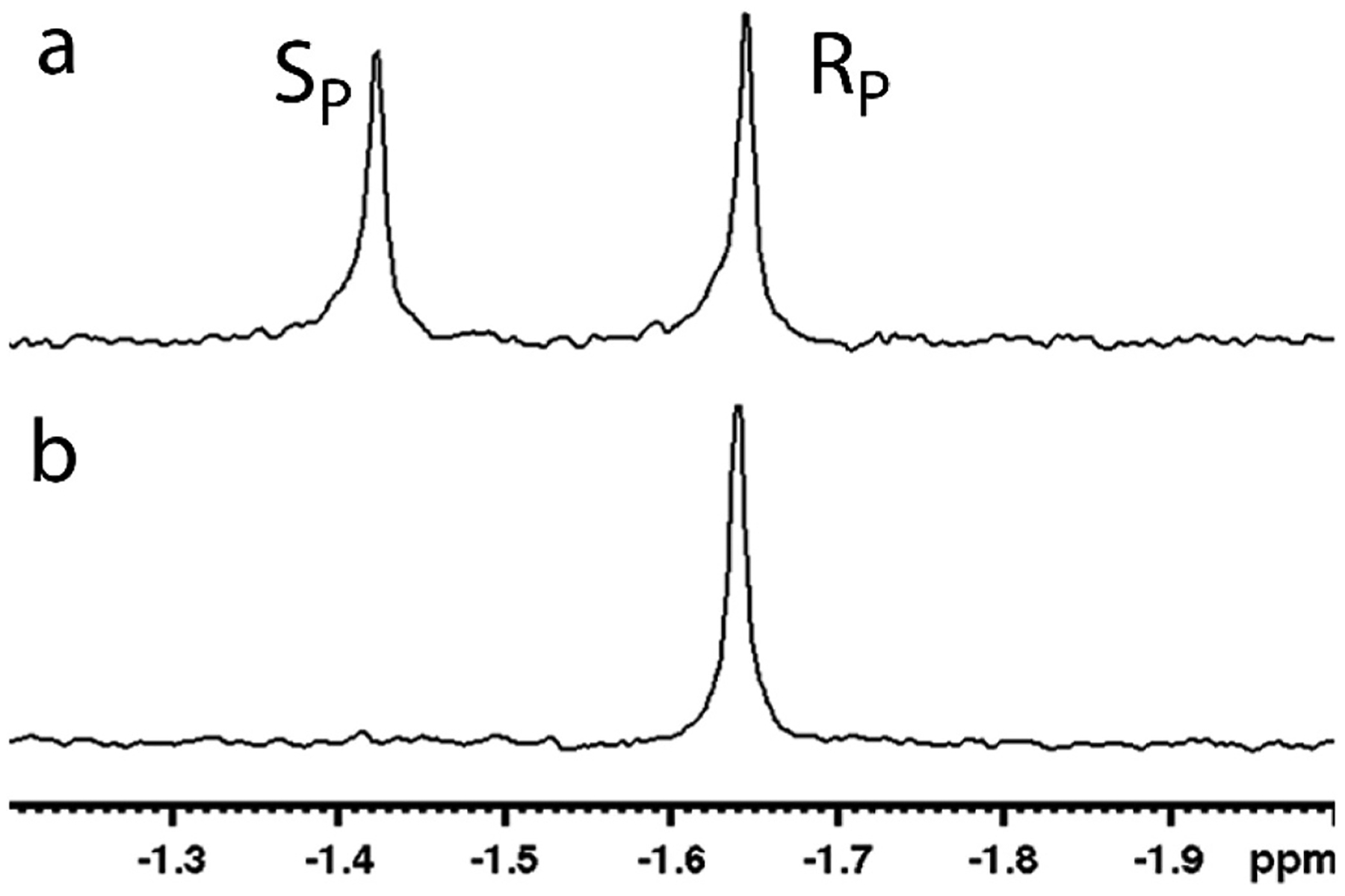
^31^P NMR of compound **2**. a) Racemic compound **2**. The R_P_-isomer resonates at −1.66 ppm. The S_P_-isomer resonates at −1.44 ppm. b) Isolated R_P_-isomer of compound **2** following selective hydrolysis by In1W-PTE. Data reproduced with permission from reference 70.

The *p*-nitrophenyl precursor to Remdesivir (compound **2**, Fig. 4) utilizes alanine esterified to a 2-ethyl butanol group. This adds considerable bulk to the amino acid group and makes selective hydrolysis more challenging for enzymes. Hydrolysis of the *p*-nitrophenyl precursors by PTE requires that the phosphoryl oxygen bind to the metal center with the leaving group in the leaving group pocket to align for the nucleophilic attack by the bridging water [58]. With the R_P_-isomers of the ProTides this places the alanine ester in the small group pocket and the phenyl group in the large group pocket (Fig. 5b). This is reversed with the S_P_-isomers which would bind with the phenyl group in the small group pocket and the alanine ester bound in the large group pocket. The variant G60A-PTE used to selectively hydrolyze the R_P_-isomer of compound **1** is well known to discriminate between phenyl and alkyl groups, but the added bulk of the amino acid ester in compound **2** rendered G60A-PTE non-selective with compound **2** [52,70]. The activity of the G60A-PTE is reduced about 10-fold with compound **2** (k_cat_/K_m_ = 4.5 × 10^2^ M^−1^s^−1^) compared to compound **1** (k_cat_/K_m_ = 2.8 × 10^3^ M^−1^s^−1^) [70]. The In1W-PTE variant was found have good activity against compound **2** (k_cat_/K_m_ = 5.4 × 10^5^ M^−1^s^−1^) and more than a 200-fold preference for the S_P_-isomer. This selectivity allowed for the isolation pure R_P_-isomer with levels of the S_P_-isomer undetectable by ^31^P NMR (Fig. 8) [70]. The pure precursor was then successfully utilized to synthesize pure *R*_P_-Remdesivir without the need for additional protecting groups. The kinetic constants are summerized in Table 1.

#### 4.3.2. Evolution of PTE for Remdesivir synthesis

Numerous variants of PTE with differing substrate preferences and stereoselectivity were screened for the ability to selectively hydrolyze R_P_-compound **2** [70]. Unfortunately, none of the tested variants proved appropriate for the isolation of pure **S**_**P**_**-2** making the production of S_P_-Remdesivir impossible by a chemoenzymatic synthesis. To overcome this challenge Uengwetwanit and co-workers used the program Triangle Matcher to computationally dock compounds **1** and **2** in the active site of PTE [71]. This allowed the identification of points of contact between the protein and ligand and allowed rational design of select variants that might improve the activity against the R_P_-isomer. They then constructed and tested variants of PTE designed to increase the size of the small group pocket (I106A, L303A, and L308A), decrease the size of the large group pocket (H254Y and L271F) and modify the leaving group pocket (W131 M) as well as numerous other variants at these positions to potentially alter the binding of **R**_**P**_**-2**. Somewhat surprisingly, modification to the large and small group pockets only had modest effects on the activity against **R**_**P**_**-2**. Modification of W131 in the leaving group pocket had a dramatic effect on the ability to hydrolyze the R_P_-isomer of compound **2**. This mutation increased the catalytic efficiency for **R**_**P**_**-2** by ~3000-fold and resulted in nearly 200-fold selectivity for the R_P_-isomer. W131 is not an amino acid that has been found to be critically important for stereoselectivity in previous studies [52,64,69]. W131 was identified as part of the leaving group pocket, but lies adjacent to the small group pocket. It appears that reducing the bulk of this residue has opened the active site to allow the binding of the larger amino acid ester of compound **2** in the small group pocket. The effect was further amplified by combination with the small group pocket I106A. The variant I106A/W131M-PTE shows more than 300-fold selectivity for the *R*_P_-isomer of compound **2** effectively allowing the isolation of the pure *S*_P_-isomer. The kinetic constants are provided in Table 1.

### 4.4. Advantage of PTE in the preparation of ProTides

The production of ProTides by the addition of the phosphonamidate group generally results in a racemic mixture of the final product [20,31]. As has been seen, the activity of the isomers can differ significantly in terms of tissue distribution and activation by the native esterases in the cell [11,23,24,33]. In many cases this is advantageous as a compound can be targeted to the tissues effected by a particular virus, but in other cases like COVID-19 which effects a wide array of tissues this problematic [4,11,18,32,72,73]. It is important in the testing and development of the ProTides that both isomers be evaluated for activity against an array of viruses in multiple tissues. This has been challenging and in some cases the isomer chosen is the one that is easiest to prepare [20]. The chemical means of obtaining the ProTides as single isomers require the elimination of one of the isomers of the precursor and harsh conditions that can require additional protecting groups to the ProTide to prevent unwanted side products due to the need to include excess of the phosphonamidate group [40,46]. The use of PTE to produce optically pure single isomers of the phosphonamidate precursor has significant advantages. This methodology effectively eliminates the undesired isomer prior to coupling to the nucleoside core producing only the desired isomer in the final product [70]. The hydrolyzed isomer can potentially be recycled to the racemate as is seen in other systems further eliminating waste. Probably the greatest advantage of this method is the ability to generate either isomer of ProTides to allow the facile testing of both compounds. As has now been demonstrated, the phosphonamidate of Sofosbuvir containing the isopropyl ester and that of Remdesivir containing the larger 2-ethyl butanol ester can be easily isolated to yield either isomer of the final ProTide [70,71]. Other ProTides such as Rovafovir etalafenamide contain a smaller ethyl ester which can almost certainly be separated by the described variants of PTE.

## 5. Future directions for the use of PTE enzymes in ProTide development

There are significant advances in the field of ProTide development that will likely benefit from the application of phosphotriesterase enzymes in the future. These include the use of phosphinamidates with a carbon phosphorus bond to improve circulation time, the use of long chain fatty acid esters to yield ultra long-lasting pharmacokinetics, and the application of ProTides to biosensing applications.

### 5.1. Application of phosphotriesterase enzyme to phosphinamidate ProTides

ProTides like Sofosbuvir and Remdesivir contain phosphonamidate triesters to mask the charge on the phosphate. These groups are added to the nucleoside via a simple S_N_2 like substitution by a nucleophilic attack of the nucleoside hydroxyl on the phosphorus liberating the labile group in the precursor [31,40,46]. Other ProTides, like Tenofovir alafenamide and Rovafovir etalafenamide, utilize a phosphinamidate group which contains a carbon phosphorus bond and the nucleoside is linked to the phosphorus through an ether bond [22,74]. The phosphinamidate group of Rovafovir etalafenamide can be synthesized as a precursor, but the linkage to the nucleoside is via the alcohol of the phosphonate after activation of the nucleoside for nucleophilic attack [43,75]. The precursor used in Rovafovir etalafenamide is purified to a single isomer by chiral chromatography or crystallization [43]. Conversely Tenofovir alafenamide is synthesized by addition of the phosphorus to the nucleoside analog before introduction of the amino acid ester and the final product must be purified to a single isomer by crystallization [74,76]. It is quite likely that optically pure ProTides could be obtained by enzymatic kinetic resolution of either the precursor or the final racemic ProTide, but this would require the hydrolysis of the phenyl group from the phosphinamidate. The PTE from B. diminuta shows approximately 5-orders of magnitude less activity with a phenyl leaving group compared to a p-nitrophenyl group suggesting that PTE may not be practical for the phosphinamidate ProTides [77]. However, the phosphotriesterase from Sphingobium sp. TCM1 (Sb-PTE) is known to readily hydrolyze phenyl groups at rates comparable to p-nitrophenyl groups [78]. The best-known substrate for Sb-PTE is triphenyl phosphate indicating that Sb-PTE is quite capable of hydrolyzing larger substrates. Sb-PTE was tested in for the ability to hydrolyze compound **1**, and it was found that with the S_P_-isomer the phenyl group was preferentially hydrolyzed over the p-nitrophenyl group demonstrating that Sb-PTE is capable of hydrolyzing the phenyl groups from phosphonamidates [67]. While it remains to be tested, it appears that *Sb*-PTE could readily be adapted for the kinetic resolution of the phosphinamidate ProTides to yield pure isomers either at the precursor or final product stage.

### 5.2. Development of long acting ProTides

There are significant efforts to develop long and ultra long-acting ProTides to treat chronic viral infections such as hepatitis and HIV. These efforts are focused on including larger and more hydrophobic groups on the phosphonamidate group. Changing the amino acid in Tenofovir alafenamide to a phenylalanine and esterification to a medium chain alcohol resulted in months long suppression of hepatitis B [79,80]. The inclusion of the medium chain alcohol (1-docosanol) has the added benefit that the alcohol has antiviral effects against enveloped viruses [81]. Similar efforts are underway with the ProTides of Abacavir and Lamivudine [82–84]. Resolution of these considerably larger ProTides will likely be more challenging, but variants of PTE such as the In1W-PTE and I106A/W131 M will likely still be useful in the kinetic resolution to allow isolated isomers [70,71].

### 5.3. Adaption of ProTide technology to biosensing and imaging

One of the more interesting applications of ProTide based technology has been proposed by Kamiya, Urano and co-workers [85]. They have adapted the basic idea to the phosphonamidate used in the ProTides to detect specific cells that express carboxy peptidases. In this work they have linked a fluorophore to a tert-butyl phosphonate along with an alanine-arginine or alanine-glutamate dipeptide. While attached to the phosphorus the fluorophore is non-fluorescent. When the peptide is cleaved by a carboxy peptidase the free carboxyl of the alanine forms the typical cyclic intermediate resulting in the release of the fluorophore and a detectable signal. The carboxy peptidases being targeted are ones known to be activated in cancers which would allow this technology to identify cancerous cells. The developed molecules contain a chiral phosphorus center. As has been seen with [26] carboxylesterase 1 and cathepsin A, enzymes that cleave these compounds are likely to be affected by the stereochemistry at the phosphorus center. Isolating the individual stereoisomers would likely lead to better selectivity for the desired peptidase. While this would need to be tested, it is likely that amongst the many variants of PTE that have been generated over the years that variants could be identified which will stereo-selectively hydrolyze these biosensing molecules.

## Figures and Tables

**Table 1:** Values of *k*_cat_/*K*_m_ for the hydrolysis of compounds **1** and **2** by phosphotriesterase.

Substrate	Enzyme	*K*_cat_*/K*_m_ (M^−1^ S^−1^)	Reference
*R*_P_ **-1**	wild-type	(4.7 ± 0.1) × 10^4^	67
*R*_P_ **-1**	G60A	(2.8 ± 0.1) × 10^3^	67
*R*_P_ **-1**	In1W	(1.6 ± 0.2) × 10^2^	67
*S*_P_ **-1**	wild-type	(7.4 ± 0.1) × 10^3^	67
*S*_P_ **-1**	G60A	(1.7 ± 0.1) × 10	67
S_P_**-1**	In1W	(2.3 ± 0.1) × 10^5^	67
*R*_P_ **-2**	G60A	(4.5 ± 0.1) × 10^2^	70
*R*_P_ **-2**	In1W	<1.0 × 10^3^	70
*R*_P_ **-2**	I106A/W131 M	(3.9 ± 0.5) × 10^4^	71
*S*_P_ **-2**	G60A	(4.5 ± 0.1) × 10^2^	70
*S*_P_ **-2**	In1W	(5.4 ± 0.8) × 10^5^	70
*S*_P_ **-2**	I106A/W131 M	(1.2 ± 0.2) × 10^2^	71

## Data Availability

Data will be made available on request.
